# Whole genome amplification and its impact on CGH array profiles

**DOI:** 10.1186/1756-0500-1-56

**Published:** 2008-07-29

**Authors:** Bente A Talseth-Palmer, Nikola A Bowden, Alyssa Hill, Cliff Meldrum, Rodney J Scott

**Affiliations:** 1 School of Biomedical Sciences, Faculty of Health, University of Newcastle and the Hunter Medical Research Institute, NSW, Australia; 2 Division of Genetics, Hunter Area Pathology Service, John Hunter Hospital, Newcastle, NSW Australia

## Abstract

**Background:**

Some array comparative genomic hybridisation (array CGH) platforms require a minimum of micrograms of DNA for the generation of reliable and reproducible data. For studies where there are limited amounts of genetic material, whole genome amplification (WGA) is an attractive method for generating sufficient quantities of genomic material from miniscule amounts of starting material. A range of WGA methods are available and the multiple displacement amplification (MDA) approach has been shown to be highly accurate, although amplification bias has been reported. In the current study, WGA was used to amplify DNA extracted from whole blood. In total, six array CGH experiments were performed to investigate whether the use of whole genome amplified DNA (wgaDNA) produces reliable and reproducible results. Four experiments were conducted on amplified DNA compared to unamplified DNA and two experiments on unamplified DNA compared to unamplified DNA.

**Findings:**

All the experiments involving wgaDNA resulted in a high proportion of losses and gains of genomic material. Previously, amplification bias has been overcome by using amplified DNA in both the test and reference DNA. Our data suggests that this approach may not be effective, as the gains and losses introduced by WGA appears to be random and are not reproducible between different experiments using the same DNA.

**Conclusion:**

In light of these findings, the use of both amplified test and reference DNA on CGH arrays may not provide an accurate representation of copy number variation in the DNA.

## Background

Comparative genomic hybridisation (CGH) was developed to detect deletions, duplications and amplifications in genomic DNA by producing a map of DNA sequence copy number against its chromosomal location [1]. This process can require several micrograms of DNA and in situations where there are limited quantities of genomic DNA (gDNA) available for experimentation the utility of whole genome amplification (WGA) is very attractive for genetic studies.

The multiple displacement amplification (MDA) WGA method replicates the genome isothermally using random hexamer primers and DNA polymerase (e.g. Phi29) followed by strand-displacement [2]. Studies on Phi29 have reported almost complete genome coverage with little amplification bias and high accuracy [2,3], which is thought to be due to the high-quality proof reading activity of the enzyme [4]. Nevertheless, amplification bias has been reported [5,6], which may be caused by Phi29 replicating one chromosome preferentially in the initial stages of the reaction [7]. It also appears that the method does not replicate highly repetitive centromeric regions effectively [8]. High concordance rates and reproducibility in single nucleotide polymorphism (SNP) genotyping studies have been reported between whole genome amplified DNA (wgaDNA) and gDNA [5,9-11] but controversial results have been reported [12]. There is in addition data to suggest that WGA creates imbalanced amplification of alleles resulting in mistyping of heterozygote genotypes as homozygotes [5,13], which is a result of unequal efficiency in the amplification of the two alleles.

The uniformity of chromosome coverage of WGA via the MDA method has been tested using CGH arrays, and a significant amplification bias between different genomic sequences, particularly at the ends of chromosomes, has been reported [14]. Lage *et al*. [14] suggests that this problem can be overcome by using wgaDNA in both reference and test sample, while others have overcome the problem by using additional statistical methods to avoid exclusion of genomic regions affected by amplification distortion and high variability [15]. It has also been demonstrated that wgaDNA compared to gDNA did not induce significant amplification bias when compared by quantitative PCR, SNP genotyping, southern blotting, restriction fragment length polymorphism (RFLP) analysis and CGH [3], even though there was an indication of loss of the repetitive centromeric regions.

Because of the necessity for the use of wgaDNA in our project, and the inconsistency between results reported on wgaDNA and CGH arrays, whole genome CGH experiments were conducted utilising commercial microarrays from Spectral Genomics to determine the effect of using wgaDNA compared to gDNA.

## Methods

### DNA samples

Human female and male pooled gDNA (Promega) was used to optimise the CGH method. DNA was extracted from whole blood from 2 controls (healthy female and male) and 1 patient in remission from acute lymphoblastic leukaemia (ALL) by the salt-precipitation method [16].

### Whole genome amplification (WGA)

Multiple displacement amplification (MDA) was performed using the GenomiPhi kit (GE Healthcare Life Sciences) and fragmentation, adaptor-ligation PCR (FLP) was performed with GenomePlex (Rubicon Genomics Inc.) according to the manufacturer's instructions (see protocols in additional file 1), with minor variation; the ethanol precipitation step at the end was omitted, as the DNA was purified with a DNA Clean and Concentrator kit (Zymo Research) before use on the CGH arrays.

### Quantitation of DNA samples

Quant-iT™ DNA assay kit (Molecular Probes Inc) was used to quantify the concentration of the DNA samples, both before and after WGA, using a fluorometer: Fluostar Optima (BMG Labtech Pty Ltd).

### Comparative genomic hybridisation (CGH)

The SpectralChip2600™ array consisted of 2632 BAC clones positioned at approximately 1 Mb intervals throughout the genome. DNA samples were fragmented twice using a Branson Sonifier 250 (Branson Ultrasonics Corporation), before purification with a DNA Clean and Concentrator™ kit (Zymo Research). DNA labelling with Cy3- and Cy5-dCTP (Amersham Biosciences) was performed using the BioPrime Labelling Kit (Invitrogen). DNA labelling, hybridisation and washing of the arrays was performed as per manufacturers' instructions (see protocol in additional file 1). Dye-reversal experiments with reciprocal labelling of the test and reference DNA were performed for each experiment. A clone was called abnormal/significant only if observed on both hybridisations. The arrays were scanned using GenePix 4000B scanner (Axon Instruments, Union City, CA) and the images analysed using GenePix Pro 3.0 software. The result files were analysed using Spectralware™ version 2.0 (Spectral Genomics). The software converts two-colour fluorescent dye signals into intensity ratio profiles. The different experiments conducted are described in Table 1.

**Table 1:** Explanation of which samples are analysed in the different experiments.

**Experiment #**	**Description**	**Amount of gDNA used in WGA reaction**	**Yield of WGA products (wgaDNA)**
Experiment 1	gDNA (Promega): Female pool vs. male pool		
Experiment 2	Female control: wgaDNA vs. gDNA	200 ng	2520 ng
Experiment 3	Female control: wgaDNA vs. gDNA	200 ng	3470 ng
Experiment 4	Male control: wgaDNA vs. gDNA	200 ng	3870 ng
Experiment 5	Male control: wgaDNA vs. gDNA^1^	200 ng	3900 ng
Experiment 6	Male ALL gDNA vs. male control gDNA^2^		

wgaDNA is DNA amplified using GenomiPhi unless otherwise noted.

^1 ^DNA is amplified using GenomePlex.

^2 ^ALL patient, DNA extracted from whole blood.

## Results

To validate and optimise the CGH array method, female and male pooled DNA samples were analysed using the X chromosome as an internal standard. The data was normalised by global linear regression and the threshold of significance of ratios was between 0.7–0.8 and 1.2–1.5 in all experiments. Any clones outside these values were considered deleted or duplicated if observed in both hybridisations (dye-swap experiments). The database of Genomic Variants [17] was used to identify common polymorphisms and single clones were considered clinically relevant.

**Figure 1:**
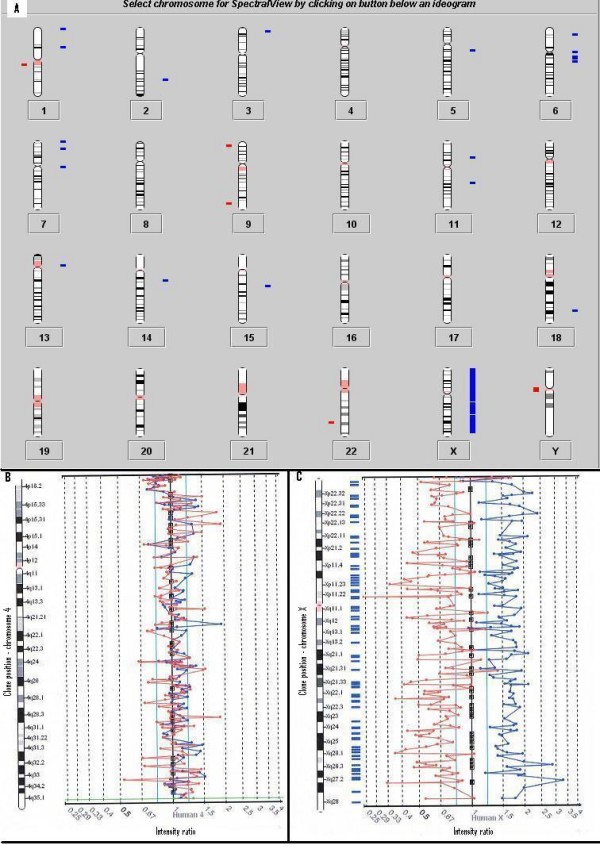
Experiment 1: The two colour fluorescent dye signals are converted into intensity ratio profiles. The upper and lower ratio threshold is 1.2 and 0.8 respectively. **A) **Ideogram showing all chromosomes. Differences seen between the female and male control (pooled gDNA from Promega) are illustrated. Gains of the X chromosome are clearly present as well as 21 duplicated and 6 deleted clones. Bars to the right (blue) indicate gains and to the left (red) losses. **B) **Chromosome 4 ratio plot. No deleted or duplicated clones on chromosome 4. **C) **Chromosome X ratio plot, a complete gain of the X chromosome can be seen.

The observed number of significant clones gained and lost can be seen in Table 2 and are listed in additional file 2. Experiment 1 (pooled female vs. pooled male) showed complete gain of the X chromosome in the female pooled DNA sample as well as 21 duplicated and 6 deleted clones (see Figure 1). All the significant clones were single clone alterations, except for two locations, 6q14 (RP11-343P23 and RP11-79L15) and 7p21.1 (cosIIIA0 and IH3).

**Table 2:** Observed numbers of significant clones gained and lost in a dye swap experiment.

**Experiment #**	**Description**	**Gain (n)**	**Loss (n)**
Experiment 1	gDNA (Promega): Female pool vs. male pool	21	6
Experiment 2	Female control: wgaDNA vs. gDNA	20	120
Experiment 3	Female control: wgaDNA vs. gDNA	1	69
Experiment 4	Male control: wgaDNA vs. gDNA	17	134
Experiment 5	Male control: wgaDNA vs. gDNA^1^	11	59
Experiment 6	Male ALL gDNA vs. male control gDNA^3^	7	3

wgaDNA is DNA amplified using GenomiPhi unless otherwise noted.

^1 ^DNA is amplified using GenomePlex.

^2 ^ALL patient, DNA extracted from whole blood.

**Figure 2:**
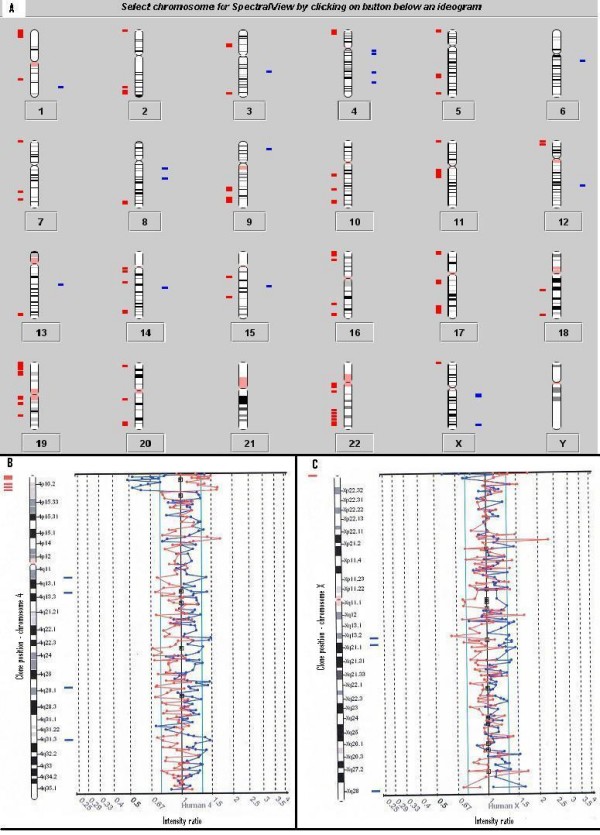
Experiment 4: The two colour fluorescent dye signals are converted into intensity ratio profiles. The upper and lower ratio threshold is 1.3 and 0.7 respectively. **A) **Ideogram showing all chromosomes. Differences seen between the amplified DNA and the non-amplified DNA in the male control are illustrated. 17 duplicated and 134 deleted clones were detected. Bars to the right (blue) indicate gains and to the left (red) losses.**B) **Chromosome 4 ratio plot. A deletion can be seen at chromosome 4p16 and duplicated single clone alterations at location 4q13.1, 4q13-4q21, 4q28.1 and 4q32. **C) **Chromosome X ratio plot. A deletion at location Xp22.33 and duplications at Xp21.1-21.31, Xp21.1-21.32 and Xq28 can be seen.

For an initial evaluation of the wgaDNA, CGH arrays were performed using female control wgaDNA vs. female control gDNA. The control female gDNA sample underwent two independent WGA reactions using the same method (GenomiPhi). The first CGH experiment (experiment 2) resulted in 20 duplicated and 120 deleted clones, while the second CGH experiment (experiment 3) produced 1 duplicated and 69 deleted clones. Only 23 of the significant clones were the same in both experiments (colour marked in additional file 2). The experiment was repeated with male control gDNA, using two different WGA methods (GenomiPhi and GenomePlex), and similar results were obtained. The first CGH experiment (experiment 4) resulted in 17 duplicated and 134 deleted clones (see Figure 2), while the second CGH experiment (experiment 5) revealed 11 duplicated and 59 deleted clones. Only 16 clones were the same in both experiments (colour marked in additional file 2).

**Figure 3:**
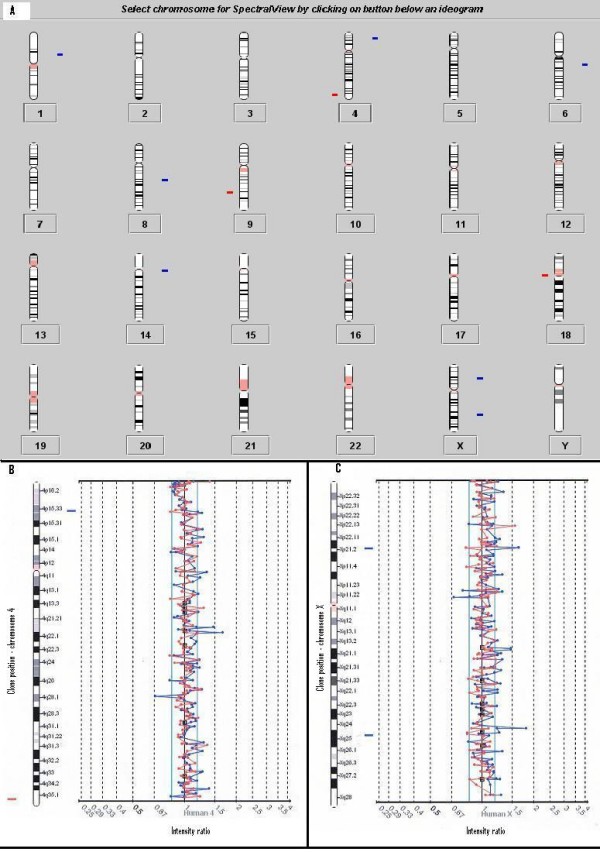
Experiment 6: The two colour fluorescent dye signals are converted into intensity ratio profiles. The upper and lower ratio threshold is 1.2 and 0.8 respectively. **A) **Ideogram showing all chromosomes. Differences seen between a male ALL patient vs. male control (both non-amplified DNA) are illustrated. 7 duplicated and 3 deleted clones were detected. Bars to the right (blue) indicate gains and to the left (red) losses. **B) **Chromosome 4 ratio plot, one clone duplication at 4p15.3 and one clone deletion at 4q35.2 can be seen. **C) **Chromosome X ratio plot shows two duplicated clones at locations Xp21.1 and Xq25.

To investigate whether the observed results could be due to procedural (CGH method) or analytical problems (SpectralWare), salt extracted gDNA from whole blood from a male patient in remission from ALL vs. gDNA from normal male control was tested. The results of this CGH experiment yielded only 7 duplicated and 3 deleted clones (see Figure 3), all single clone alterations.

## Discussion

The quality of wgaDNA has been primarily tested using SNP genotyping to check the concordance between unamplified and amplified DNA. It has been shown that there is little, if any, degradation in the accuracy of SNP genotyping with wgaDNA compared to gDNA [5]. Tzvetkov *et al*. [13] performed SNP array analysis using 4 samples, and showed that there was good concordance between wgaDNA and gDNA, although the percentage of called SNPs in wgaDNA samples was lower than that observed in the gDNA. A study by Paez *et al*. [10] estimated that 99.82% of the genome was correctly replicated by WGA but six regions (1q42, 4q35, 6p25, 7q36, 10q26 and 18p11) were consistently underrepresented in wgaDNA and a further eight regions were questionable due to low signal intensity. It has also been observed that the MDA method results in an amplification bias that can misrepresent the true number of heterozygous genotypes [7]. The loss of heterozygous genotypes is due to one allele being preferentially amplified in the early stages of MDA. A certain amount of sequence bias is to be expected with any WGA method, which may result in variation in DNA quality used in the reaction, GC content, repetitive sequences or priming efficiency. The generation of aspecific products both in the presence of non-human DNA and even when no DNA template is present has been observed [14,15,18].

Sun *et al*. [19] reported that in WGA products, allelic imbalance is common when the starting material is less than 1 ng and rarely occurs with starting material over 5 ng. Only small amounts of input material (5 ng) are required for amplification using the GenomiPhi method and gives an average yield of approximately 2 μg [7]. In the current study, 200 ng of DNA was used for the WGA reaction of the controls and was expected to yield reproducible results, as previous studies testing the MDA method have also utilised high concentrations of starting material [20,21], but this was not our experience. However, insufficient amounts of DNA is a more commonly reported cause of poor WGA [19].

The current study demonstrates that wgaDNA does not result in reproducible results when compared to gDNA from the same sample. Of the 25 variations observed in experiment 1, 24 were in regions that have been reported to contain a variation in a healthy control population, indicating that they are most likely common copy number variations. Copy number variation in a pool of DNA from different donors are expected to be cancelled out and if single clone alterations are not deemed significant, this could therefore be the case for experiment 1. For the purpose of this study, we find it necessary to report single clone alterations as the clinical significance of them is unknown. In experiments 2–5 most of the significant clones were single clone alterations, suggesting that the observed gains and losses are relatively small in size, but there was low reproducibility in the chromosomal locations of the observed gains and losses.

In experiments 4 and 5 two different WGA methods were compared, which creates a possible bias, as these two methods might not be directly comparable. The MDA method amplifies fragments of 70 kb from the original DNA [7], therefore it can be argued that the sonication step is unnecessary and a source of variation of the signal ratio. Gel electrophoresis of both sonicated gDNA and wgaDNA showed no obvious size difference (data not shown), this should therefore not induce the observed alterations. Recently, it has been reported that direct labelling of the amplified DNA is preferred over random primed labelling, which involves an additional amplification of the product [22]. The additional amplification step could cause bias and thus be a source of observed alterations. Consequently, the use of random prime labelling in this study may have led to the unusually high number of observed alterations. As the GenomiPhi method produced high numbers of irreproducible gains and losses, we found it necessary to test another WGA method, but similar results were observed. In experiment 6 an ALL sample were tested. DNA extracted from children in remission from ALL was available for this project, so we tested the WGA method on one of these samples. At this stage of the study, we solely wanted to confirm that the CGH platform is providing reproducible and reliable results in the absence of wgaDNA, which it was.

The lack of reproducibility in the experiments in this study was somewhat surprising since previous studies have produced favourable results. For example, Dean *et al*. [3] compared wgaDNA to gDNA from the same sample, CGH to chromosome spreads, and found that WGA does not induce significant amplification bias. However, the sensitivity of this approach would not be accurate enough to detect differences that are identified by array CGH, because the resolution of CGH is lower than array CGH. The majority of array CGH studies have compared test wgaDNA to reference wgaDNA [14,20,23], which is the most likely explanation for the differing results. Hughes *et al*. [23] have shown, by utilising Spectral Genomics BAC arrays, that WGA does not introduce any major distortion of imbalance of gDNA when using CGH arrays. They performed control experiments corresponding to DNA before and after amplification (gDNA vs. gDNA and wgaDNA vs. wgaDNA) on the same sample. They also demonstrated that DOP-PCR introduced a number of additional copy number aberrations, while MDA introduced no detectable bias [24]. In contrast, we demonstrated that gDNA vs. wgaDNA results in bias that was different between two experiments using the same DNA sample, suggesting the random nature of WGA has to be taken into account when considering this method for array CGH analysis. Lage *et al*. [14] evaluated DNA amplification bias by assessing two experiments on the same amplified samples: female gDNA vs. male gDNA and female wgaDNA vs. male wgaDNA. Although the same number of autosomal data points (n = 20) was observed outside the confidence limits for both experiments [14], there could be up to 40 differences in deletions/duplications detected since the chromosomal locations of the observed differences in each experiment were not reported. A number of WGA methods are available and although it is commonly reported that WGA introduces sequence bias, the methods are still widely used as a lot of projects only have finite amounts of DNA available.

In conclusion, WGA appears not to be an ideal method for increasing DNA yields for use on CGH arrays in our laboratory as it creates an unacceptable amount of deletions and duplications that are not reproducible between experiments. In light of these findings, the use of both amplified test and reference DNA on CGH arrays may not provide an accurate representation of copy number variation in DNA.

## Competing interests

The authors declare that they have no competing interests.

## Authors' contributions

BAT-P carried out the experimental procedures, data analysis and interpretation and drafted the manuscript. NAB contributed to the design of the experiments, data analysis and revision of the manuscript. AH participated in the experimental procedures. CM participated in the design of the study and revision of the manuscript. RJS conceived of the study, participated in its design and coordination, and helped to draft and revise the manuscript. All authors read and approved the final manuscript.

## Supplementary Material

Additional file 1 Protocols. Protocols for GenomiPhi, GenomPlex and SpectralChip 2600, each method has its own excel sheet. Click here for file

Additional file 2 Results. Lists of significant clones in all experiments, each experiment has its own excel sheet. Click here for file
